# Large cities get more for less: Water footprint efficiency across the US

**DOI:** 10.1371/journal.pone.0202301

**Published:** 2018-08-20

**Authors:** Tasnuva Mahjabin, Susana Garcia, Caitlin Grady, Alfonso Mejia

**Affiliations:** 1 Department of Civil and Environmental Engineering, The Pennsylvania State University, University Park, Pennsylvania, United States of America; 2 Rock Ethics Institute, The Pennsylvania State University, University Park, Pennsylvania, United States of America; Mercator Research Institute on Global Commons and Climate Change gGmbH, GERMANY

## Abstract

Many urban indicators and functional citywide properties have been shown to scale with population due to agglomeration effects. We hypothesize that scaling relations may also exist for water-related urban indicators such as the water footprint. The water footprint is an indicator of water use that measures humans’ appropriation of freshwater resources. We analyze the scaling of the water footprint for 65 mid- to large-sized US cities using both empirical estimates and a social interaction network model of city functioning. The network model is used to explain the presence of any scaling exponent in the empirical estimates of the urban water footprint by linking to previous theories of urban scaling. We find that the urban water footprint tends to approximately show sublinear scaling behavior with both population and gross domestic product. Thus, large cities tend to be more water footprint efficient and productive than mid-sized cities, where efficiency and productivity are quantified, in a broad sense, as deviations from a linear scaling exponent. We find the sublinear scaling may be linked to changes in urban economic structure with city size, which lead to large cities shifting water intensive economic activities to less populated regions. In addition, we find that green water contributes to the scaling both positively by transferring the dependence of food consumption on population into the water footprint and negatively by increasing heterogeneity. Overall, the proposed scaling relations allow for the comparison of water footprint efficiency and productivity of cities. Comparing these properties and identifying deviations from the expected behavior has implications for water resources and urban sustainability.

## Introduction

For the first time in human history, the 21^th^ century has seen the advent of a city-dominated human settlement pattern where the majority of people now live in cities [1]. People are moving to cities because of opportunities, economic development, change in social structure and human behavior [2]. By 2030, cities in developing countries are expected to double in population and cities in developed countries are expected to increase by 20% [3]. It is increasingly recognized that cities are central to global sustainability because they can collectively create substantial stress on interconnected natural resources (e.g., food and water) [4] and have governance structures that allow for more flexible and independent decision making [5]. Urban indicators are commonly used to track and assess urban sustainability [6]. In order to explore the ability of cities to achieve sustainability goals and targets, it is important to quantitatively understand how such indicators may vary among cities.

Recently, it has been shown that many urban indicators and functional citywide properties can be quantified mathematically through scaling relationships. According to scaling theory, several specific properties of cities vary on average with their size in predictable ways [7]. Robustness in scaling exponents has been found for a wide variety of urban indicators which makes scaling a pervasive feature of urban systems [8]. Studies have shown that there are per capita increases (superlinear scaling) in socioeconomic indicators such as Gross Domestic Product (GDP), employment, invention rate, etc., as a function of population size, indicating that larger cities are more prosperous, innovative and productive [9, 10]. In contrast, variables related to material infrastructure, e.g., the length of electric cables and road surfaces, exhibit economies of scale as they increase slower than the city population size (sublinear scaling) [11, 12]. Linear scaling with population has been associated with basic human needs such as household consumption of water and electricity, housing, and jobs [8]. Several studies have also found that scaling relationships exist for urban environmental indicators, e.g., CO_2_ emissions [13–16].

A wide variety of disciplines such as engineering [17], economics [12, 18], complex systems [11, 19, 20] and geography [19, 21] have demonstrated that many characteristics of cities can be quantified and predicted mathematically due to agglomeration or scaling effects. Recently, Bettencourt [11] proposed a theoretical framework to predict the scaling exponents of different urban quantities using the properties of social and infrastructural networks. According to his theory, the functional properties of cities are determined by the interactions of the population embedded in a massive social network. These social connections, which are also part of other networks such as transportation, electrical, communications, etc., mainly control how people, things, and information interact across urban space and time. In a different explanation of urban scaling, Gomez-Lievano et al. [22] proposed that urban scaling depends on the level of economic complexity and therefore scaling phenomena will occur when all the necessary complimentary factors are simultaneously present [20, 22]. In this study, we use Bettencourt’s network-based theory to analyze empirical results for the scaling of water-related urban indicators.

“Living system” or “organism” are often used as metaphors of urban functioning since cities, much like an organism, consume and produce resources, goods, services, and information. This view of cities is central to urban metabolism which can be defined as “the sum total of the technical and socio-economic processes that occur in cities, resulting in growth, production of energy, and elimination of waste” [23, 24]. Urban metabolism has tended to emphasize the direct flow of resources and materials through an urban system [24–26]. To expand this framework, in the context of water resources, water footprints can be used to consider indirect flows. A water footprint is an indicator of consumptive water use that accounts for the direct water flows that enter a geographic area as physical water and the indirect water flows, also known as virtual water flows, embedded in the consumption of goods and services [27–31]. The virtual or embedded water is an important component of water footprints because it provides information about the dependency of a geographic region on distant water resources [32]. Previous studies have estimated water footprints at the national or global level [29, 30, 33, 34] and a few studies have also focused on regional or city level [32, 35–40]. A main focus of city-level water footprint studies has been on quantifying direct and indirect water uses [41], highlighting that water can be saved at a much broader scale by targeting a city’s indirect water use such as the water consumed through commodity production and consumption [38, 39]. In contrast, our focus here is in the scaling behavior of the urban water footprint.

In this study, our primary objective is to analyze the scaling of the water footprint with population for 65 mid- to large-sized US cities. The scaling is analyzed using both empirical and theoretical estimates. The latter are obtained using a social network model of city functioning. The model is used to explain the presence of any scaling exponent in the urban water footprints by linking to previous theories and mathematical relations of urban scaling. To determine the urban water footprints, we account for the direct and indirect water used by cities in the production and consumption of food and industrial commodities. Our motivation for examining the scaling of urban water footprints is to understand whether large cities are more water footprint efficient and to explain deviations from and the likely sources of any efficiency. In this study, we define urban water footprint efficiency broadly as deviations (sublinear or superlinear) from linearity in the values of urban water footprint scaling exponents. In the case of a sublinear scaling exponent (exponents less than 1), we denote the urban water footprint scaling as “efficient” since the urban water footprint per capita decreases with increasing population size, i.e., each urbanite has a lesser water footprint as the city size increases. In the case of superlinear scaling (exponents greater than 1), the scaling exponent is used to denote inefficiency since each urbanite has a greater water footprint as city size increases. This way of measuring urban water footprint efficiency is mainly concerned with economic-related efficiencies (e.g., allocation of resources, economic specialization, comparative advantages, etc.), as opposed to technological-related (e.g., drip versus spray irrigation) or policy-related (e.g., improved reservoir operations) efficiencies. Ultimately, understanding the scaling behavior of the urban water footprint may be practically useful to devise strategies and policy intended to enhance urban water sustainability, e.g., through cross city comparisons and by fostering greater water resources use accountability.

## Materials and methods

### Data

The urban water footprint was computed using agricultural, livestock and industrial commodity flows, and their corresponding virtual water contents, *VWC*. For the commodity flows, we used 2007 Freight Analysis Framework version 3 (FAF3) data [42]. The FAF3 data represents, for the year 2007, the flow of economic commodities among 123 different origin-destination regions encompassing the entire geography of the US. Out of these 123 regions, 73 are metropolitan statistical areas (MSAs). MSAs are defined by the US Census Bureau as a geographical region containing at least one city core with population greater or equal 50,000, together with any adjacent counties that have a strong economic tie to the city core. After combining the 6 FAF3 MSAs that have boundaries overlapping multiple states, we ended up with 65 MSAs. We used these 65 MSAs to compute and assess the scaling behavior of the urban water footprint. These 65 MSAs account for the largest and some of the major mid-sized US cities. Notice that to expand the number of cities considered would require modeling the entire commodity trade network of any additional city not included in the FAF3 data. This is deemed outside the scope of the present analysis. We only accounted for US cities because of data availability, i.e., the FAF3 data only includes US cities.

The FAF3 data also contains commodity flows between the US and various international regions, specified as import and export flows. Only export flows were included since our analysis focuses on US cities. The FAF3 data divides the entire US product economy into 43 different commodity classes. Out of these 43 commodity classes, we included 6 agricultural and livestock classes (hereafter food classes) and 24 industrial classes. Only the commodity classes related to the energy and mining sectors were left out.

To transform the FAF3 commodity flows into virtual water flows, we used *VWC* data from previous studies [32, 33, 43]. We estimated the *VWC* of the different FAF3 food commodity classes using *VWC* data for individual crops and livestock from Mekonnen and Hoekstra [33] and Mubako [43], production data from the US Department of Agriculture (USDA) [44], and by applying the averaging approach of Dang et al. [45]. The *VWC* of the different industrial commodity classes were obtained from the study by Ahams et al. [32], which relied on available estimates of volume of water used per employee for individual industries at the US national level [46]. The *VWC* data for the food commodities accounts for both blue and green water. The blue water accounts for the consumptive use of water originating from ground or surface water sources [30] while the green water accounts, in this case, for the rainwater consumed as evapotranspiration in agricultural production [33]. Using the commodity flow and *VWC* data, the water footprint of production, *WFP*, and consumption, *WFC*, were determined as follows

$$WFP_{i}=\sum_{j}\sum_{k}VWC_{i,k}T_{i\to j,k},\;\mathrm{and}$$ (1)

$$WFC_{i}=\sum_{j}\sum_{k}VWC_{j,k}T_{j\to i,k}+\Delta_{i},$$ (2)

respectively, where *i* indexes different cities. In Eq (1), *VWC*_*i*,*k*_ is the virtual water content of commodity *k* in the FAF3 city *i*, and *T*_*i*→*j*,*k*_ is the tonnage of *k* produced by city *i* and consumed by FAF3 region *j*. Note that region *j* can be a city or non-city region. In Eq (2), *VWC*_*j*,*k*_ is the virtual water content of commodity *k* in the FAF3 region *j*, and *T*_*j*→*i*,*k*_ is the tonnage of *k* produced by the FAF3 region *j* and consumed by city *i*. The term Δ_*i*_ in Eq (2) denotes the consumptive use of domestic and commercial water [47, 48] by city *i*. These consumptive water uses were obtained from the USGS as in Ahams et al. [32]. The urban water footprint, *WF*, of city *i* was determined as follows

$$WF_{i}=WFC_{i}+WFP_{i}.$$ (3)

**Fig 1:**
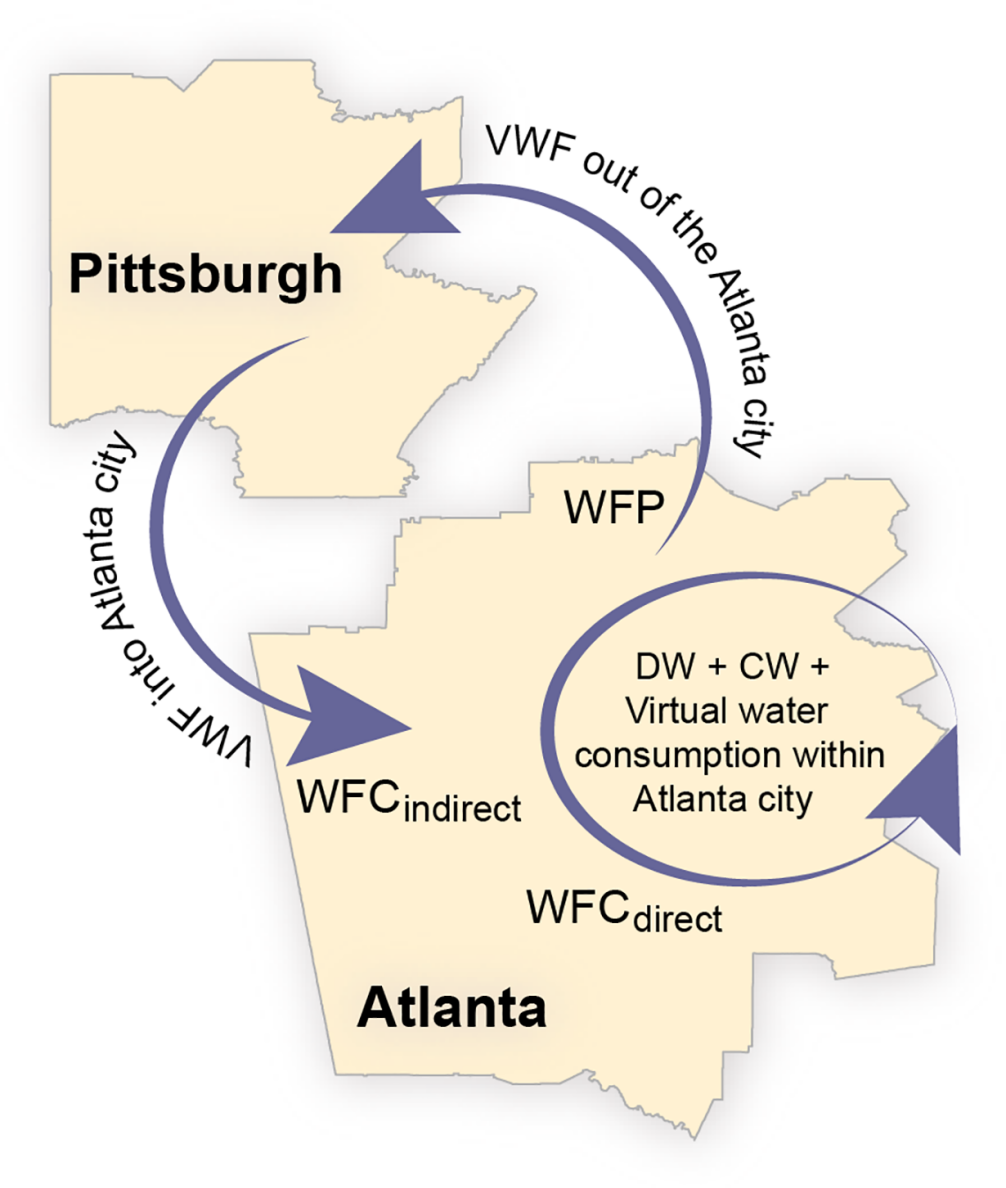
Illustration of hypothetical virtual water flows (*VWF*) between two cities: Atlanta and Pittsburgh. The meaning of the variables is as follows: water footprint of consumption, *WFC*; water footprint of production, *WFP*; domestic water, *DW*; and commercial water, *CW*.

*WFC* involves both direct and indirect consumptive water uses, whereas *WFP* only considers direct uses. Direct consumptive water uses occur within city boundaries while indirect consumptive water uses take place outside city boundaries. Indirect consumptive water uses are virtually transferred to a city through the consumption of food and industrial commodities. For example, in the hypothetical illustration in Fig 1, the direct *WFC* of Atlanta city (Fig 1) is equal to the water used for domestic and commercial activities as well as the water used to produce food and industrial commodities for self-consumption. The indirect *WFC* of Atlanta consists in Fig 1 of the virtual water transferred through the consumption of food and industrial commodities produced in Pittsburg. In terms of production, *WFP* accounts for the direct water used to produce food and industrial commodities in Atlanta that are then consumed in Pittsburg (Fig 1).

### Scaling analysis

Urban scaling implies that urban indicators exhibit self-similarity [49]. The self-similarity of an urban indicator can be expressed as follows [8]

$$Y(N,t)=Y_{o}(t)N{\left(t\right)}^{\beta }e^{\xi (t)},$$ (4)

where *Y*(*N*, *t*) is an urban indicator or citywide property that depends on the population *N* at time *t*, *Y*_*o*_(*t*) is a baseline prefactor common to all cities at time *t*, *β* is a dimensionless scaling exponent, and *ξ*(*t*) are statistical fluctuations that account for deviations from the expected value. At a fixed time, with *ξ*(*t*) being Gaussian, Eq (4) takes the following form in logarithmic space

$$\ln Y_{i}=\ln Y_{o}+\beta \ln N_{i}+\xi_{i}.$$ (5)

Thus, in Eq (5), *β* is the slope and ln*Y*_*o*_ the intercept of the line implied by the regression of *Y* on *N*. With *Y* and *N* given by the data, the coefficients *β* and ln*Y*_*o*_ can be obtained by the method of ordinary least squares minimization. The scaling exponent *β* is of particular interest as it provides information about the behavior of the indicator *Y*. For instance, it has been shown that when *Y* is urban infrastructure (e.g., the length of transportation infrastructure), *β* tends to be sublinear, *β* < 1, and when *Y* is equal to an urban socioeconomic output (e.g., GDP), *β* is superlinear, *β* > 1. We used Eq (5), together with our water footprint estimates from Eqs (1)–(3), to empirically assess the scaling of the urban water footprint with population. In this study we performed scaling analysis with both population and GDP where population data was collected from US Census [50] for the year 2007 and GDP was obtained from Bureau of Economic Analysis (BEA) [51].

## Results

### Scaling of urban commodity consumption and production

We begin the scaling analysis by revisiting the social network model of urban scaling proposed by Bettencourt [11]. Following from a few basic principles, the model can be used to obtain, in a unified and quantitative way, theoretical scaling exponents for different urban indicators and functional properties. Our aim is to first use the model to obtain likely, theoretical scaling exponents for *WFC* and *WFP*, and then compare those values against the empirical scaling exponents obtained from the data. To implement the network model and derive the theoretical exponents, we rely on several previous scaling and mathematical relations used to explain urban indicators [11]. At the end, however, we obtain a single theoretical exponent that depends only on two variables. The derivation of this theoretical exponent is shown next.

To implement the social network model, we let *Y* in Eq (5) be equal to the food or industrial commodities consumed or produced by a city in terms of monetary value. *Y* is expressed first in terms of monetary value to facilitate the implementation of the model. Later, we convert *Y* from units of monetary value to units of volume of water to determine the scaling exponents for *WFC* and *WFP*. The network model assumes the scaling is due to a citywide network of social interactions set, on average, by the population density over the total area of the network, *N*/*A*_*n*_ [11]. Furthering this analysis, we suggest that, instead of *N*/*A*_*n*_, the relevant network of interactions is in this case set by *N*_*f*_/*A*_*n*_, where *N*_*f*_ is the fraction of population *N* interacting in the social network associated with food or industrial commodities. *N*_*f*_/*A*_*n*_ is used because we only account for the fraction of the urban economy associated with food and industrial commodities, as opposed to accounting for all the economic activities of a city. The interactions *N*_*f*_/*A*_*n*_ are translated into urban outputs, either outputs for individual consumption or production, using the following [11]

$$\frac{Y}{N_{f}}=J\frac{N_{f}}{A_{n}},$$ (6)

where *Y*/*N*_*f*_ is the per individual output and *J* is a constant that translates the interactions per individual into urban outputs.

Under short-term spatial economic equilibrium, the net socioeconomic benefit per capita, *Y*/*N*_*f*_, is set equal to the transportation or mobility costs *C*. The costs are represented, as often done in urban and regional economics [52, 53], by the distant *L* such that *C*~*L*. The length, in turn, can be related in a very general way to the area of the city by *L*~*A*^*H*/*D*^ [11], where *A* is the city area, *D* sets the appropriate spatial dimension (*D* = 2 in this case since one is dealing with an area), and *H* is the fractal dimension. The simplest assumption is to set *H* = 1 [10] which means that individuals can fully explore the city area within the shortest distance traveled. With the previous relationships for *C* and *L*, and setting *Y*/*N*_*f*_ equal to *C*, we have that

$$\frac{Y}{N_{f}}∼A^{H/D}.$$ (7)

For the total network area *A*_*n*_, the following relationship is adopted [11]

$$A_{n}∼{\left(\frac{A}{N_{f}}\right)}^{1/D}N_{f},$$ (8)

where the term (*A*/*N*_*f*_)^1/*D*^ is obtained by setting the average distance between individuals equal to the average length of infrastructure network per capita. The form of Eq (8) is motivated by previous findings [8]. Using *A* instead of *A*_*n*_ in Eq (6) to represent the per capita output in terms of city area, and combining Eqs (6) and (7), we get *A*~*N*_*f*_^*α*^, where *α* = *D*/(*D*+*H*). The scaling relationship *A*~*N*_*f*_^*α*^ is substituted into Eq (8) to obtain *A*_*n*_~*N*_*f*_^1−*δ*^, where *δ* = *H*/[*D*(*D*+*H*)]. Lastly, substituting the latter scaling relationship into Eq (6), we obtain

$$Y∼N_{f}{}^{1+\delta }.$$ (9)

To empirically evaluate Eq (9), *N*_*f*_ needs to be known but there are no datasets available that relate the urban consumption or production of food and industrial commodities to *N*_*f*_. Therefore, as a proxy to *N*_*f*_ and to gain tractability, the number of establishments *N*_*b*_ associated with economic sector or industry *b* is used, since previous results have shown that *N*_*b*_~*N*^*γ*^ where the scaling exponent *γ* varies with the type of industry and, more broadly, with the economic sector (primary, secondary, or tertiary) [12]. Thus, letting *N*_*f*_~*N*^*γ*^ and substituting into Eq (9), one has that

$$Y∼N^{\gamma (1+\delta )}.$$ (10)

Contrasting Eqs (4) and (10), the theoretical value of the scaling exponent in Eq (10) is *β* = *γ*(1+*δ*) or $\beta =\frac{7}{6}\gamma$ with *D* = 2 and *H* = 1.

Although we have relied on several equations, Eqs (6)–(9), to obtain a theoretical expression for the exponent *β*, hereafter we only use Eq (10) to explain any empirical scaling exponents for the urban water footprint. By including the exponent *γ* in Eq (10), we have modified the original theoretical exponent obtained by Bettencourt [11] to account for the effect of different economic industries on the scaling. When taking into consideration all the economic industries in a city, however, one expects *γ* = 1 [12] so that the scaling exponent of Bettencourt [11], namely 1+*δ*, is recovered. Since *γ* can vary over a wide range of values [12], from 0 to 1.2, the theoretical exponent *β*, in this case, may be sublinear or superlinear depending on the value of *γ*.

**Fig 2:**
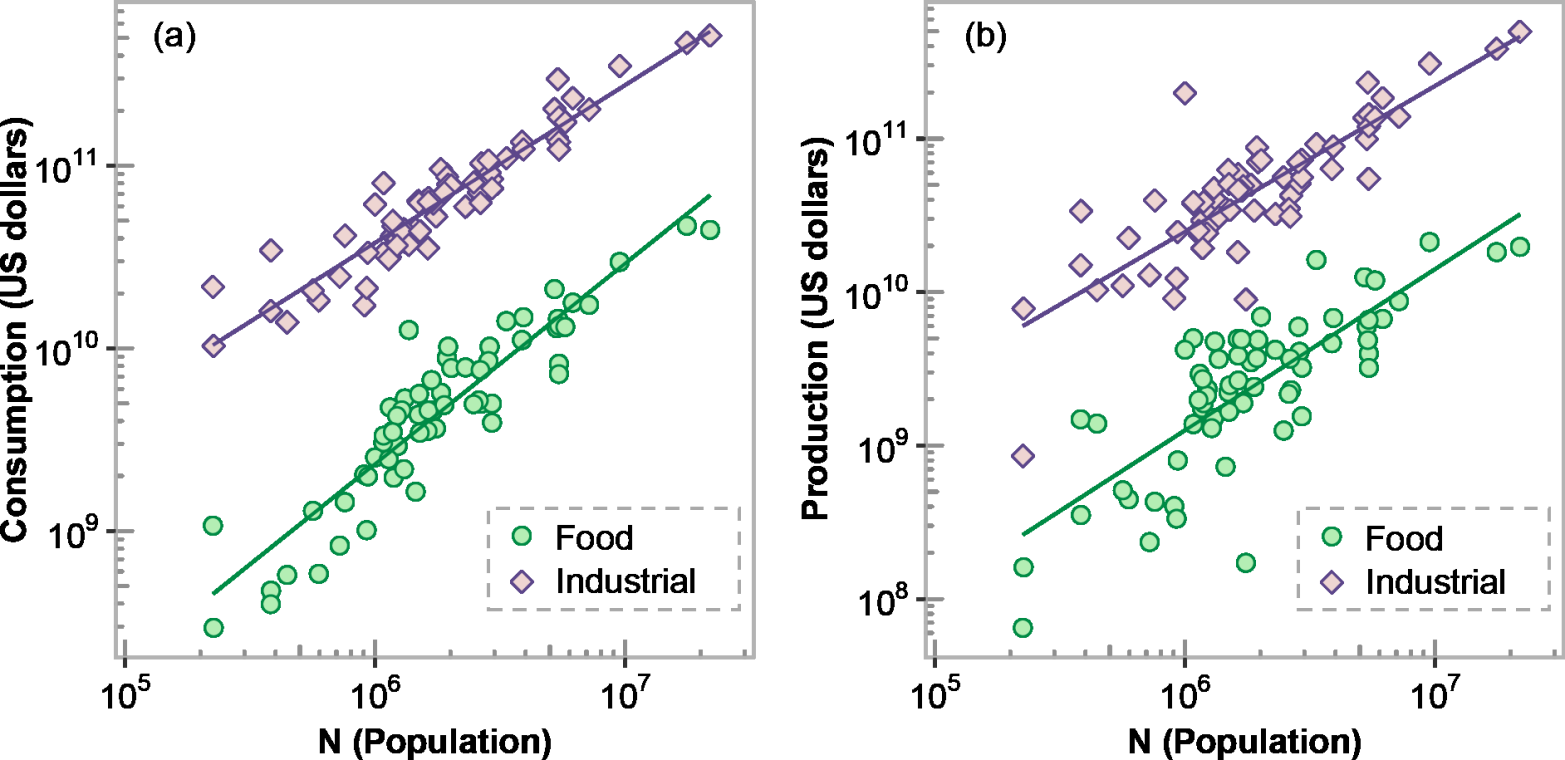
(a) Urban scaling of commodity consumption (expressed in monetary value) with population size. For food commodities, *β* = 1.10 (95% CI [0.98, 1.22]) and *R*^2^ = 0.84. For industrial commodities, *β* = 0.86 (95% CI [0.79, 0.94]) and *R*^2^ = 0.89. (b) Urban scaling of commodity production (expressed in monetary value) with population size. For food commodities, *β* = 1.05 (95% CI [0.85, 1.25]) and *R*^2^ = 0.63. For industrial commodities, *β* = 0.95 (95% CI [0.79, 1.11]) and *R*^2^ = 0.70. For all cases, *p*-value<0.001 and the line indicates the best-fitted line by ordinary least squares in logarithmic scale.

Based on the results of Youn et al. [12], the value of *γ* for food commodities is likely between 0.9 (food services) and 1.0 (manufacturing), which implies according to Eq (10) that the theoretical value of *β* is approximately in the range 1.05–1.17. We found by fitting Eq (5) to the data that the empirical value of *β* is 1.10 (*R*^2^ = 0.84, *p*-value<0.001) and 1.05 (*R*^2^ = 0.63, *p*-value<0.001) for urban food commodity consumption (Fig 2A) and production (Fig 2B), respectively. Thus, the theoretical and empirical values of *β* compare well against each other. Youn et al. [12] do not report individual values of *γ* for the industry classes considered in the FAF3 data. They, however, found the value of *γ* decreases from approximately 1.2 to 0 for industries in the service and primary sector, respectively, with secondary sector industries having values of *γ* in between 1 and 0. Also, the industrial commodities in the FAF3 data consist of a heterogeneous mix of mostly secondary sector industries, which are less prevalent in large cities and likely to engage a smaller fraction of *N* than the food commodities. Because of these considerations, it is reasonable to expect for the industrial commodities that the value of *β* is close to or somewhat less than 1 (i.e., *γ*<0.85). We found the empirical values of *β* to be 0.86 (*R*^2^ = 0.89, *p*-value<0.001) and 0.95 (*R*^2^ = 0.70, *p*-value<0.001) for urban industrial commodity consumption (Fig 2A) and production (Fig 2B), respectively. These values of *β* imply that *γ* is equal to 0.74 and 0.81, respectively, which corresponds with our general observation that *γ*<0.85. Hereafter we omit reporting the *p*-value since it is always less than 0.001.

Notice that the value of *β* is sublinear for both urban industrial commodity consumption and production. This is due to the value of *γ* being less than 0.85 for both cases. This differs from the sublinear scaling associated with infrastructure efficiencies [11], which applies when the scaling variable under consideration is urban infrastructure. Thus, in this case, the value of *β* varies depending on the economic industries considered and the change in the prevalence of those industries with urban population, i.e., the exponent *γ*.

### Scaling of the urban water footprint of consumption and production

To link the previous theoretical exponents to the scaling exponent for *WFC*, we added urban food and industrial commodity consumption together to obtain the total urban commodity consumption *Y*_*c*_. The scaling exponent *β*' in *Y*_*c*_~*N*^*β*'^ was obtained as the weighted average of the scaling exponents for food, *β* = 1.10, and industrial, *β* = 0.86, consumption following the approach in S1 Appendix. The approach allows to approximate the scaling exponent of the sum of two scaling variables [7]. *β*' is employed here, as opposed to *β*, to indicate that the scaling exponent for *Y*_*c*_ is approximated from two other more fundamental scaling exponents. Using the weighted average approach, we found *β*' = 0.86, which compares well with the empirical value of 0.87 (*R*^2^ = 0.90) estimated directly from the data. Notice that the weighted average approach allows to track the effect of *β* on *β*' because of this, hereafter, the exponents obtained in this manner are referred to as semi-theoretical.

To transform *Y*_*c*_ from units of monetary value to volume of water, we used the dependence between *V*_*c*_ and *Y*_*c*_, where *V*_*c*_ denotes the virtual water flows associated with total urban commodity consumption. By setting Δ = 0 in Eq (2), one simply has that *V*_*c*_ = *WFC*, which is a reasonable assumption because the magnitude of virtual water flows in Eq (2) is much greater than the value of Δ [32]. The dependence between *V*_*c*_ and *Y*_*c*_ can be generally described by *V*_*c*_~*Y*_*c*_^*ϕ*^. The reason for this is that *V*_*c*_ is expected to scale with population because as urban population increases so does total food consumption and, in consequence, virtual water flows. Virtual water flows increase with population mainly because of green water, which rises as the amount of cropland consumption grows. Therefore, through the mutual dependence of *V*_*c*_ and *Y*_*c*_ on population, we expect the two variables to relate to each other. Indeed, we found in this case that *ϕ* = 1.02 (*R*^2^ = 0.68). Combining *Y*_*c*_~*N*^*β*'^ and *V*_*c*_~*Y*_*c*_^*ϕ*^, and letting *V*_*c*_ = *WFC*, we found that the semi-theoretical scaling exponent for *WFC*~*N*^*ϕβ*'^ is approximately given by *ϕβ*' = 0.88. A similar analysis was performed for *WFP*. We found that *WFP*~*N*^*ϕβ*'^ with *β*' = 0.95 (using the weighted average approach), *ϕ* = 0.93 (*R*^2^ = 0.58), and *ϕβ*' = 0.88, with the empirical estimate for *β*' being 0.95 (*R*^2^ = 0.71). Interestingly, the value of *ϕ* is relatively close to 1 for both *WFC* and *WFP*, thus having only a mild effect on the scaling. Table 1 summarizes the relationships and exponents used to obtain the scaling exponents for *WFC* and *WFP*.

**Table 1:** Summary of scaling exponents used to explain the scaling of *WFC* and *WFP*.

Variables	Exponents	
	Empirical	Weighted average
Total (food and industrial) urban commodity consumption in monetary value versus population, *Y*_*c*_~*N*^*β*'^	*β*' = 0.87	*β*' = 0.86
Total urban commodity production in monetary value versus population, *Y*_*p*_~*N*^*β*'^	*β*' = 0.95	*β*' = 0.95
Total urban commodity consumption in units of volume of virtual water versus the same variable in units of monetary value, *V*_*c*_~*Y*_*c*_^*ϕ*^	*ϕ* = 1.02	-
Total urban commodity production in units of volume of virtual water versus the same variable in units of monetary value, *V*_*p*_~*Y*_*p*_^*ϕ*^	*ϕ* = 0.93	-
Water footprint of consumption versus population, *WFC*~*N*^*ϕβ*'^	*ϕβ*' = 0.92	*ϕβ*' = 0.88
Water footprint of production versus population, *WFP*~*N*^*ϕβ*'^	*ϕβ*' = 0.91	*ϕβ*' = 0.88

**Fig 3:**
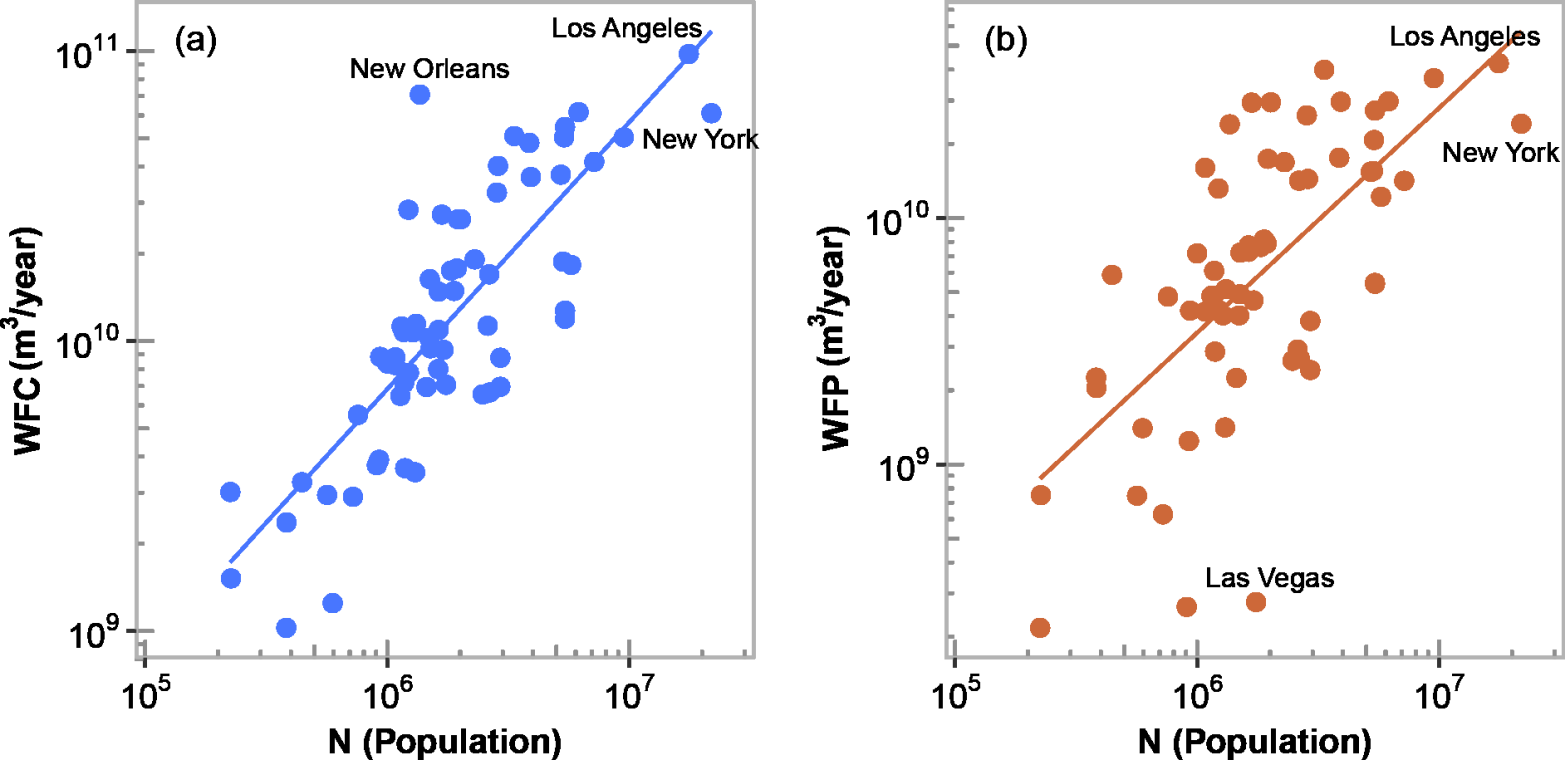
(a) Scaling of the water footprint of consumption (*WFC*) with population size where the scaling exponent is 0.92 (95% CI [0.75, 1.09]) and *R*^2^ = 0.65. (b) Scaling of the water footprint of production (*WFP*) with population size where scaling exponent is 0.91 (95% CI [0.66, 1.17]) and *R*^2^ = 0.44. For all cases, *p*-value<0.001 and the line indicates the best-fitted line by ordinary least squares in logarithmic scale.

The semi-theoretical scaling exponent *ϕβ*' for *WFC* and *WFP*, which is equal to 0.88 in both cases, is relatively close to the empirical estimates of 0.92 (Fig 3A) and 0.91 (Fig 3B), respectively. Thus, both *WFC* and *WFP* exhibit slightly sublinear scaling with population, indicating that large cities tend to be more water footrpint efficient than mid-sized cities, based on our definition of water footprint efficiency, *β*<1. It is, however, apparent in Fig 3 that the scaling is only approximate as there is substantial variability in the plots and some cities (e.g., New Orleans in Fig 3A and Las Vegas in Fig 3B) show large deviations from the expected value. Also, *WFP* shows more heterogeneity than *WFC* (Fig 3). This is not surprising since the internal economic structure of cities varies from city to city as they specialize in different economic sectors, and some cities (e.g., New Orleans has an unusually high *WFP* value) may be specializing in more water intensive activities. In addition, *WFC* has much higher values than those of *WFP*. For example, Los Angeles has a *WFC* value of 9.8 x 10^10^ m^3^/year (Fig 3A), which is more than twice its *WFP* value (Fig 3B). We elaborate in the discussion section on the sources of variability in the approximate scaling of *WFC* and *WFP*.

We can explain the sublinear scaling of *WFC* and *WFP* using the theoretical scaling exponent obtained for *β* in Eq (10). Accordingly, the scaling of *WFC* and *WFP* depends solely on two independent scaling exponents, namely *δ* and *γ*. That is, the scaling of *WFC* and *WFP* is determined by both agglomeration effects as captured by the exponent *δ* and the internal economic structure of cities as captured by *γ*. The sublinear nature of the scaling seems largely due to the value of *γ*. For instance, setting *γ* = 1 to remove the effects of internal economic structure, and given that *ϕ*≈1, then the scaling exponent for *WFC* and *WFP* become both superlinear, approximately equal to 1+*δ*. Based on this theory, the sublinear scaling of *WFC* and *WFP* is tied to the changing composition of urban economic activities with city size, suggesting that large cities are more service oriented with less prevalence of secondary sector industries. This means that large cities have reduced water footprints by shifting water-intensive economic activities to less populated regions. This highlights that the source of the efficiency (sublinear scaling) in *WFC* and *WFP* is due to cities specializing in less water-intensive activities as population increases.

### Scaling of the urban water footprint

**Fig 4:**
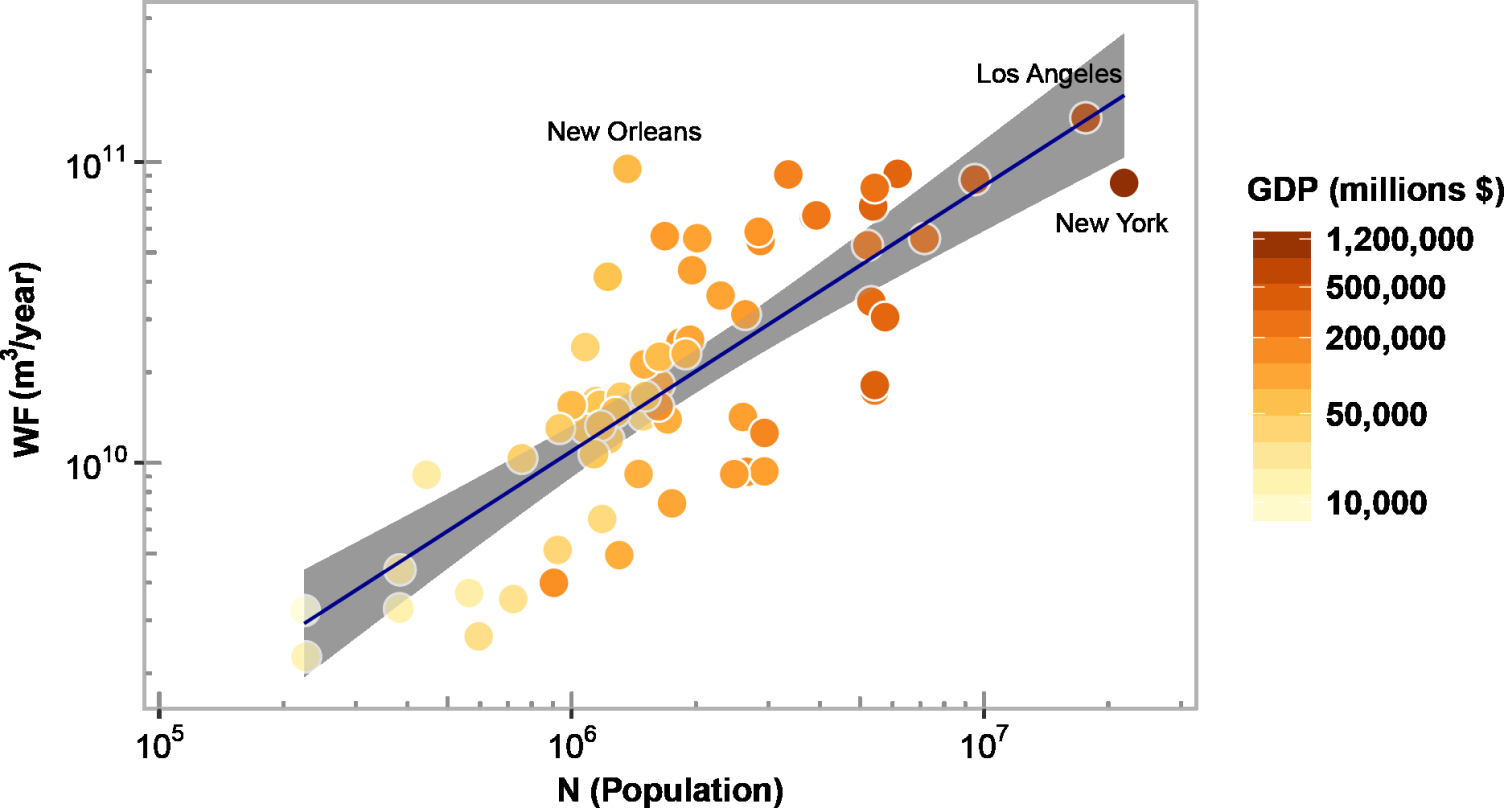
Scaling of the urban water footprint (*WF*) with population size and GDP. For *WF* vs. population, the scaling exponent is 0.88 (95% CI [0.70, 1.06]) and *R*^2^ = 0.61. For *WF* vs. GDP, the scaling exponent is 0.74 (95% CI [0.58, 0.91]) and *R*^2^ = 0.56. For all cases, *p*-value<0.001 and the line indicates the best-fitted line by ordinary least squares in logarithmic scale.

To examine the scaling of *WF* with population, we used the weighted average approach in S1 Appendix to combine the semi-theoretical scaling exponents for *WFC* and *WFP*. We found the semi-theoretical exponent for *WF* is equal to 0.88, which matches the empirical estimate (Fig 4). This indicates that the exponent for *WF* can be explained by the scaling of *WFC* and *WFP*, whose scaling exponents in turn are dependent on the values of *β*, *γ*, and *ϕ*. The data, nonetheless, show scatter in Fig 4 (*R*^*2*^ = 0.61), which was expected as our previous scaling results for *WFC* and *WFP* revealed that some cities tend to deviate from the average behavior (Fig 3). Also, when weighting the exponents for *WFC* and *WFP* to compute the semi-theoretical exponent for *WF*, we found that the weight for the *WFC* exponent was 80%, highlighting that *WFC* plays a more dominant role than *WFP* in determining the magnitude of *WF*. Hence, cities are here net virtual water importers, as also indicated by Ahams et al. [32].

The approximate scaling of *WF* is sublinear (Fig 4), as was the case for *WFC* and *WFP*, indicating that the per capita *WF* declines as the urban population increases. In other words, in largely populated US cities, each person has on average a smaller *WF* than in less populated cities. In addition, we determined the scaling of *WF* with GDP and found that it is sublinear with an empirical exponent equal to 0.74 (Fig 4 shows GDP as a function of population). This result was expected since urban population and GDP are known to scale [8]. However, in the present context, it reveals that the economic productivity of water tends to increase with GDP. In other words, large US cities use proportionately less water than mid-sized cities to achieve a similar increase in GDP.

**Fig 5:**
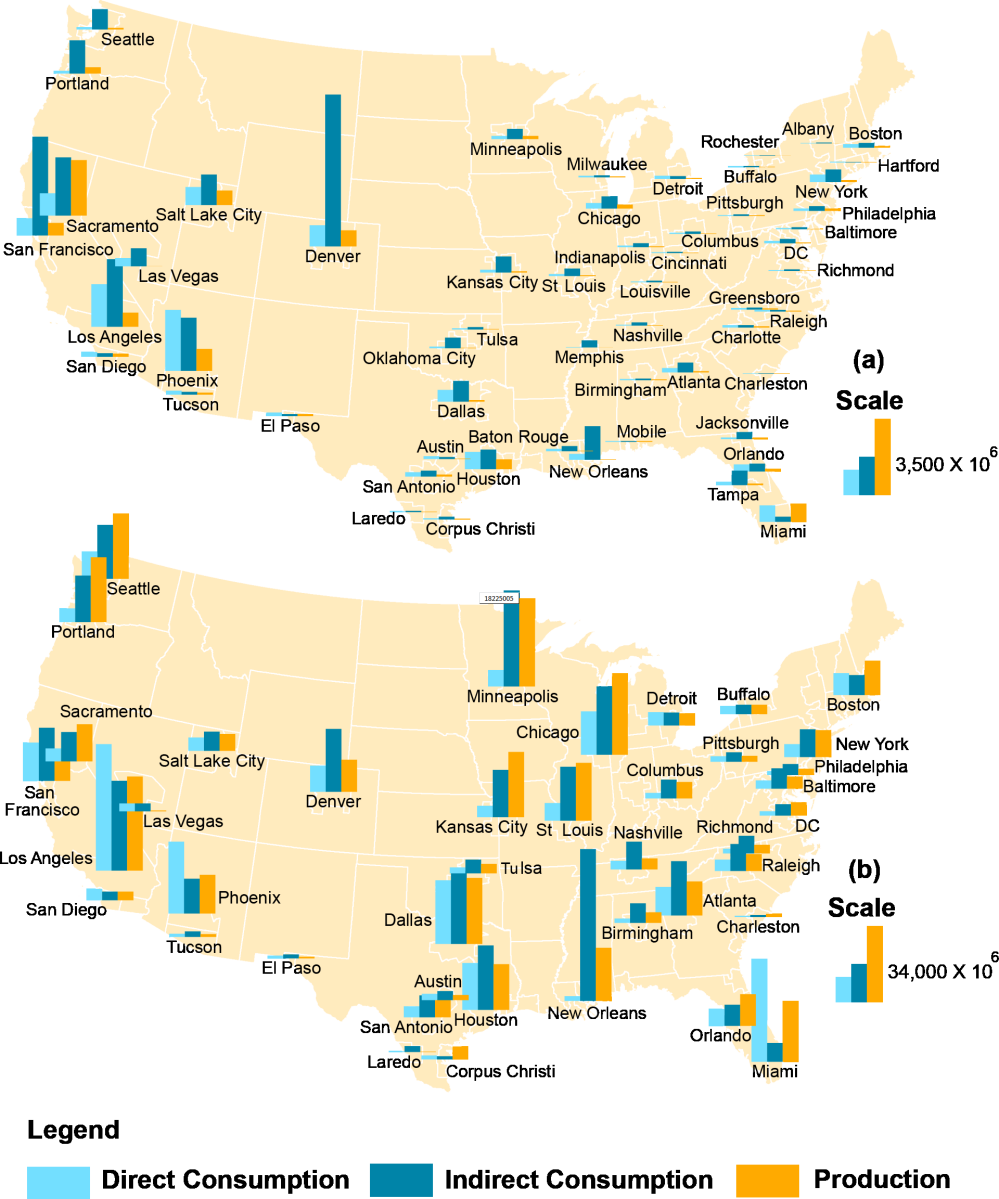
**Spatial distribution of the (a) blue water footprint (m^3^/year) and (b) blue and green water footprint (m^3^/year) of consumption and production for the analyzed US cities**. The water footprint of consumption is separated into direct and indirect contributions.

Another way of understanding the scaling of *WF* is by separating *WF* into blue and green water. We found that the blue *WF* (result not shown) shows little correlation with population and the scaling effect is therefore negligible. This is because the blue *WF* is dominated by water used to irrigate crops and green urban areas. These water use practices are much more prevalent, because of its arid and semi-arid climatic conditions, in the western half of the US than in the more humid eastern half [28]. The spatial distribution of blue *WF* for the selected cities is illustrated in Fig 5A. It is evident from this figure that western US cities are more blue *WF*-dependent than eastern cities. For example, the per capita direct blue water consumption of Phoenix is 35 times higher than that of Philadelphia.

Accounting for the combined blue and green water (Fig 5B) makes the *WF* of western and eastern cities appear more comparable. This highlights the underlying importance of green water in the approximate scaling of *WF*. This is, as mentioned before, because of the dependence of green water on population. We also separate in Fig 5 the *WF* into direct and indirect consumption. This is useful because the direct component reflects local level (within city) water requirements while the indirect component reflects regional to national level requirements. For the combined blue and green *WF*, Fig 5B shows that both direct and indirect requirements are important to city functioning. This means that cities rely equally on local and regional/national water resources to meet their economic demands for food and industrial commodities. Cities, consequently, may be considered key drivers of water security, a result that was recently highlighted for global cities [54].

## Discussion and conclusions

We have shown that large US cities tend to be more *WF* efficient than mid-sized cities, i.e., the urban *WF* tends to scale sublinearly with population. This was demonstrated using both a social network model and empirical analysis. It was also observed, however, that some cities deviate from the expected scaling behavior. Indeed, the plots of water footprint against population (Figs 3 and 4) show substantial variability around the fitted lines. Some of the scaling results from previous research for urban indicators in the US display less variability (e.g., with *R*^2^ values that are typically greater than approximately 0.85 [8]) than observed here for *WFC*, *WFP* and *WF*. A primary reason for the variability in the values of *WFC*, *WFP* and *WF* is due to these indicators being mixed quantities that depend on more fundamental scaling relationships. This becomes apparent when one compares the performance of the scaling of urban food, *R*^2^ = 0.84, and industrial, *R*^2^ = 0.89, consumption in monetary value against the scaling of *WFC*, *R*^2^ = 0.65. The scaling results in monetary value are more robust than the results for *WFC*.

The mixed nature of the scaling of *WFC*, *WFP* and *WF* results in greater variability through two sources of heterogeneity: urban commodity production and green water. Urban commodity production shows greater variability than consumption, which affects the scaling of *WFP* and ultimately *WF*. The heterogeneity of urban commodity production is not surprising as cities specialize in different industries to gain comparative economic advantages. However, other measures of urban productivity in the US such as total wages and new patents have shown more robust scaling [8, 11] than found in this study using the FAF3 data (e.g., the *R*^2^ values for the scaling of urban food and industrial production in monetary value are 0.63 and 0.70, respectively). It is likely that the FAF3 data is contributing noise to the scaling relationships as the primary purpose of the data is to track the transport of economic commodities across regions rather than to quantify the internal functioning of cities.

Green water also has an important influence on the variability of *WF* values. The role of green water on the scaling was captured through the exponent *ϕ* which allowed to convert urban consumption and production from units of monetary value to volume of water. The relationships used to determine *ϕ* (*WFC*/*WFP* versus the total urban consumption/production) have only a moderate performance with *R*^2^ = 0.68 and 0.58 for *WFC* and *WFP*, respectively. The heterogeneity induced by green water on urban scaling is not surprising as green water, driven by evapotranspiration and cropland/pasture area and characteristics, is strongly dependent on factors external to city functioning such as climate. Indeed, it is noteworthy that, despite the variability of green water values, the *WFC* and *WFP* are able to display approximate scaling behavior. This is because green water also depends on population given that virtual water flows increase with food consumption and production through the dependence of food on cultivated cropland and pasture area.

In contrast, in the case of blue water, the value of *ϕ* is negligible (blue water shows very weak correlation with population) because two cities with comparable population can have vastly different blue water requirements, depending on whether their food consumption originates from mostly rainfed or irrigated agricultural areas. For example, the following pairs of cities have comparable population but very different blue *WF* (Fig 5A): New York-Los Angeles, Baltimore-Denver, and Indianapolis-Salt Lake City. Thus, the blue *WF* mainly mirrors hydroclimatic patterns while the green *WF* contributes both heterogeneity and regularity to the scaling behavior of *WF* through its dual dependence on hydroclimatic conditions and population.

As indicated in the result section, the sublinear scaling of *WFC* and *WFP* exhibits dependency on the changing composition of urban economic activities with city size, where large cities are more service oriented with less prevalence of secondary sector industries. This allows large cities to have reduced water footprints by shifting water intensive economic activities to less populated regions. The shifting of water-intensive economic activities from large cities to less populated regions has implications for water resources and urban sustainability. For instance, at the national level such efficiency gains are likely to be substantially reduced without further interventions (e.g., technological and consumption pattern changes). We examined the effect of international food and industrial commodity imports on the sublinear scaling of *WF* by assigning imports average *VWC* values based on the national US *VWC* estimates. We found that commodity imports only have a relatively minor effect, hence they do not explain the sublinear scaling. Instead, large US cities seem to rely on other, less populated US regions to meet their indirect water needs. Such regions, when located in water scarce places like the southwestern US, could be particularly vulnerable to ongoing and future changes in climate. Any large city dependent on the commodity production from a water scarce region, in turn, may be susceptible to those water vulnerabilities. In such situations, *WF* efficiencies may not be particularly helpful and ensuring physical water use efficiencies will be critical.

Our results also indicate conditions under which urban *WF* efficiencies are likely to be beneficial. The *WF* efficiencies implied by the sublinear scaling are desirable when cities shift water-intensive activities to less populated regions that are water abundant. This is obviously advantageous to cities located in water scarce regions. However, all cities, even those located in water abundant regions, may gain from such shift by creating opportunities for urban specialization in higher (less water-intensive) economic sectors. This suggests that growing mid-sized cities need to be attentive not to outsource water-intensive economic activities to water vulnerable regions. This would be supportive of urban *WF* efficiencies that are sustainable. In the future, it could be useful to jointly consider the scaling of *WF* with metrics of water scarcity. This could help distinguish those cities that are benefitting the most from the sublinear scaling of *WF*. Such cities, in turn, could serve as examples for other cities to learn from. The sublinear scaling of *WF* can be interpreted as representing the likely or average value of *WF* that is achievable under relevant urban constraints (i.e., the constraints associated with city functioning and economic structure captured by the theoretical scaling exponent used in this study). Then, cities that lie above the fitted scaling line may be urban areas where reductions in *WF* values are realistically feasible. Moreover, it may be possible for these cities to change while sustaining urban function or remaining resilient. Although, to be practical, we recognize that this will need to be assessed on a case-by-case basis. We have highlighted some beneficial implications of the sublinear scaling of *WF*. We believe the scaling analysis of *WF* can be used to compare and benchmark cities, and potentially set realistic targets and support the development of strategies for reducing *WF*. This information could be valuable to policy makers and city planners concerned with designing economic incentives that support water sustainability.

Overall, the approximate scaling of the *WF* suggests that despite the multiple and complex socioeconomic and natural forces that drive and influence the *WF*, and despite important sources of *WF* heterogeneity, some degree of regularity emerges at the city level that helps explain the efficiency and spatial variability of *WF*. One limitation of this study is that at the time of study completion, the FAF3 database was the most recent available data despite representing the year 2007. Since publication, 2012 data are now available in the FAF4 database. Future research could use the FAF3 and FAF4 databases to explore the effects of population change over time on the scaling behavior of the urban water footprints. However, population is most often a slowly varying function of time so that differences between 2007 and 2012 are likely to have a relatively mild effect on the scaling exponents. Several previous studies have investigated the scaling behavior of cities’ energy consumption [55] and carbon dioxide emissions [13–15]. In contrast, to our knowledge, this is the first study to assess the scaling of the urban water footprint. We believe information about the urban water footprint may help governments, private companies, and non-governmental organizations prioritize and strategize about future policies to further sustainable management of limited water resources in these critical population areas.

## Supporting information

S1 Appendix Scaling exponent of the sum of two scaling variables. (DOCX)

S1 Dataset Dataset used to perform scaling analysis of urban water footprint. (DOCX)
