# Supplementary material for: Large cities get more for less: Water footprint efficiency across the US
Source: PLoS One. 2018 Aug 20;13(8):e0202301. doi: 10.1371/journal.pone.0202301 (PMC6101394; doi:10.1371/journal.pone.0202301)
Supplement: S1 Appendix — (DOCX) [file pone.0202301.s001.docx]

**S1 Appendix. Scaling exponent of the sum of two scaling variables.**

Letting $Z=X+W$, where $X=X_{o}N^{\tau}$ and $W=W_{o}N^{\upsilon}$, the scaling exponent of *Z* can be approximated as follows

$\eta\approx\frac{\tau X_{o}+\upsilon W_{o}}{X_{o}+W_{o}},$ (S1)

such that $Z=Z_{o}N^{\eta}$. The coefficients *X_o_*, *W_o_*, and *Z_o_* are different scaling prefactors. Eq. (S1) says that $\eta$ is equal to the weighted average of the exponents $\tau$ and $\upsilon$. This is useful because in the case of a mixed quantity *Z* (e.g., *WFC*, *WFP*, or *WF*) its scaling behavior may be explained by the scaling of the variables *X* and *W*. The derivation of Eq. (S1) is demonstrated elsewhere [1]. Ultimately, the quality of the approximation in Eq. (S1) will depend on both the similarity between $\tau$ and $\upsilon$, and the value of the weighting factors.

**Reference:**

1. Bettencourt L, Lobo J, Youn H. The hypothesis of urban scaling: formalization, implications and challenges. arXiv preprint arXiv:1301.5919. 2013 Jan 24.
