# Supplementary material for: Large cities get more for less: Water footprint efficiency across the US
Source: PLoS One. 2018 Aug 20;13(8):e0202301. doi: 10.1371/journal.pone.0202301 (PMC6101394; doi:10.1371/journal.pone.0202301)
Supplement: S1 Dataset — (DOCX) [file pone.0202301.s002.docx]

**S1 Dataset: Dataset used to perform scaling analysis of urban water footprint.**

**S1 Table.** Summary of the datasets.

| **Dataset** | **Source** | **DOI/Website link** |
| --- | --- | --- |
| Freight Analysis Framework version 3 (FAF3) commodity flow data for 2007 | Southworth et al. (2011) | <https://ops.fhwa.dot.gov/freight/freight_analysis/faf/> |
| Virtual water content of crops | Mekonnen and Hoekstra (2011) | <https://doi.org/10.5194/hess-15-1577-2011> |
| Virtual water content of animals | Mubako (2011) | <http://opensiuc.lib.siu.edu/dissertations> |
| Agricultural and animal production | USDA (2007) | <https://quickstats.nass.usda.gov/> |
| Water use coefficients for 198 industrial commodity classes in the U.S. | CDM-Smith (1996) | <http://www.dtic.mil/dtic/tr/fulltext/u2/a205008.pdf> |
| Virtual water content of industrial commodities | Ahams et al. (2017) | <https://doi.org/10.1111/1752-1688.12563> |
| Consumptive use of domestic and commercial water | Kenny et al. (2009) | [http://pubs.er.usgs.gov/publication/cir1344](http://pubs.er.usgs.gov/publication/cir1344 ) |
|  | Solley et al. (1998) | [http://pubs.er.usgs.gov/publication/cir1200](http://pubs.er.usgs.gov/publication/cir1200 ) |
| Population | U.S. Census (2007) | <https://www.census.gov/library/publications/2010/>  compendia/databooks/ccdb07.html |
| GDP | BEA (2007) | <https://www.bea.gov/regional/> |

**S2 Table.** State level virtual water content (VWC) data in m³/year/tonnage for 6 food classes. Food classes are based on Standard Classification of Transported Goods (SCTG) classification system and are indicated in the parentheses.

| **State** | **Live animals and live fish (1)** | **Cereal grains (2)** | **Other agricultural products (3)** | **Animal feed and products of animal origin, n.e.c (4)** | **Meat, fish, seafood, and their preparations (5)** | **Milled grain products and preparations and bakery products (6)** |
| --- | --- | --- | --- | --- | --- | --- |
| Alabama | 3508 | 771 | 3255 | 3491 | 3498 | 798 |
| Alaska | 7039 | 987 | 130 | 6513 | 7155 | 1072 |
| Arizona | 12919 | 1220 | 1353 | 12865 | 12961 | 1381 |
| Arkansas | 3051 | 1061 | 2570 | 3034 | 3046 | 1344 |
| California | 7318 | 1211 | 506 | 7208 | 7323 | 1551 |
| Colorado | 7787 | 1568 | 173 | 7761 | 7796 | 1662 |
| Connecticut | 6437 | 503 | 556 | 6324 | 6470 | 513 |
| Delaware | 1245 | 679 | 951 | 1241 | 1244 | 716 |
| Washington DC | 5479 | 987 | 1112 | 5442 | 5479 | 1072 |
| Florida | 7800 | 938 | 300 | 7755 | 7821 | 1141 |
| Georgia | 2440 | 748 | 1932 | 2427 | 2436 | 777 |
| Hawaii | 8505 | 987 | 245 | 8427 | 8517 | 1072 |
| Idaho | 7793 | 1961 | 152 | 7780 | 7806 | 2224 |
| Illinois | 4194 | 602 | 1557 | 4169 | 4194 | 602 |
| Indiana | 4054 | 551 | 1559 | 4024 | 4050 | 550 |
| Iowa | 4005 | 558 | 1617 | 3980 | 4006 | 556 |
| Kansas | 7111 | 1201 | 1790 | 7079 | 7113 | 1274 |
| Kentucky | 6153 | 635 | 1608 | 6106 | 6148 | 637 |
| Louisiana | 3882 | 838 | 1358 | 3862 | 3877 | 920 |
| Maine | 5626 | 976 | 87 | 5472 | 5637 | 1329 |
| Maryland | 1820 | 718 | 1106 | 1801 | 1812 | 752 |
| Massachusetts | 7123 | 640 | 177 | 6985 | 7305 | 672 |
| Michigan | 4539 | 586 | 509 | 4503 | 4543 | 600 |
| Minnesota | 3632 | 588 | 628 | 3608 | 3632 | 599 |
| Mississippi | 3104 | 781 | 2428 | 3090 | 3094 | 838 |
| Missouri | 5558 | 709 | 1776 | 5520 | 5558 | 726 |
| Montana | 7558 | 2163 | 162 | 7500 | 7574 | 2368 |
| Nebraska | 5952 | 732 | 1433 | 5920 | 5954 | 733 |
| Nevada | 11194 | 2486 | 162 | 11158 | 11219 | 2670 |
| New Hampshire | 9015 | 578 | 314 | 8863 | 9128 | 610 |
| New Jersey | 6602 | 621 | 416 | 6466 | 6743 | 641 |
| New Mexico | 8721 | 1575 | 1717 | 8574 | 8738 | 1716 |
| New York | 6792 | 553 | 385 | 6677 | 6823 | 584 |
| North Carolina | 3173 | 694 | 1346 | 3145 | 3170 | 713 |
| North Dakota | 5182 | 931 | 596 | 5132 | 5192 | 1023 |
| Ohio | 3833 | 579 | 1424 | 3805 | 3829 | 588 |
| Oklahoma | 6939 | 1808 | 3799 | 6869 | 6944 | 1976 |
| Oregon | 8381 | 2091 | 277 | 8335 | 8403 | 2261 |
| Pennsylvania | 3960 | 581 | 935 | 3907 | 3956 | 613 |
| Rhode Island | 6952 | 654 | 185 | 6795 | 7014 | 686 |
| South Carolina | 3551 | 651 | 1393 | 3533 | 3541 | 668 |
| South Dakota | 5287 | 883 | 1608 | 5250 | 5292 | 913 |
| Tennessee | 5604 | 747 | 2270 | 5561 | 5611 | 755 |
| Texas | 6741 | 1279 | 4632 | 6691 | 6749 | 1373 |
| Utah | 7332 | 1852 | 1020 | 7295 | 7336 | 2049 |
| Vermont | 8825 | 538 | 370 | 8634 | 8860 | 582 |
| Virginia | 4456 | 792 | 1181 | 4421 | 4453 | 833 |
| Washington | 7020 | 2020 | 261 | 6949 | 7040 | 2179 |
| West Virginia | 4520 | 614 | 604 | 4502 | 4513 | 651 |
| Wisconsin | 5769 | 540 | 661 | 5688 | 5776 | 557 |
| Wyoming | 10467 | 1354 | 123 | 10425 | 10506 | 1568 |

**S3 Table.** National level virtual water content (VWC) data for 24 industrial SCTG classes.

| **SCTG class** | **Commodity class name** | **VWC (m³/year/tonnage)** |
| --- | --- | --- |
| 9 | Tobacco products | 1.5989 |
| 13 | Non-metallic minerals, n.e.c. | 0.0008 |
| 19 | Coal and petroleum products, n.e.c. | 0.0143 |
| 20 | Basic chemicals | 0.3091 |
| 21 | Pharmaceutical products | 0.0850 |
| 22 | Fertilizers | 0.0026 |
| 23 | Chemical products and preparations, n.e.c. | 0.5490 |
| 24 | Plastics and rubber | 0.5174 |
| 26 | Wood products | 1.5015 |
| 27 | Pulp, newsprint, paper, and paperboard | 3.1238 |
| 28 | Paper or paperboard articles | 0.0489 |
| 29 | Printed products | 0.4260 |
| 30 | Textiles, leather, and articles of textiles or leather | 2.0277 |
| 31 | Non-metallic mineral products | 0.0470 |
| 32 | Base metal in primary or semi-finished forms and in finished basic shapes | 0.3346 |
| 33 | Articles of base metal | 1.7539 |
| 34 | Machinery | 1.9625 |
| 35 | Electronic and other electrical equipment and components, and office equipment | 2.4357 |
| 36 | Motorized and other vehicles (including parts) | 0.5646 |
| 37 | Transportation equipment, n.e.c. | 12.9159 |
| 38 | Precision instruments and apparatus | 3.4841 |
| 39 | Furniture, mattresses and mattress supports, lamps, lighting fittings, and illuminated signs | 1.0471 |
| 40 | Miscellaneous Manufactured Products | 0.3068 |
| 41 | Waste and scrap | 0.0022 |

**S4 Table.** Summary of the water footprint consumption (WFC) and water footprint production (WFP) of food and industrial commodities for 65 US cities which are used to estimate scaling exponents in this study.

| **City (MSA or CSA)** | **State(s)** | **Population** | **GDP (millions of dollars)** | **Direct WFC (m³/year)** | **Indirect WFC (m³/year)** | **WFP (m³/year)** |
| --- | --- | --- | --- | --- | --- | --- |
| Albany | NY | 1145666 | 41376 | 6.92E+09 | 4.31E+09 | 4.83E+09 |
| Atlanta | GA-AL | 5213661 | 290235 | 1.29E+10 | 2.46E+10 | 1.54E+10 |
| Austin | TX | 1452529 | 79654 | 2.88E+09 | 4.06E+09 | 2.25E+09 |
| Baltimore | MD | 2655675 | 147791 | 1.74E+09 | 4.91E+09 | 2.69E+09 |
| Baton Rouge | LA | 756998 | 39929 | 1.15E+09 | 4.41E+09 | 4.79E+09 |
| Beaumont | TX | 383530 | 18798 | 6.89E+08 | 1.68E+09 | 2.05E+09 |
| Birmingham | AL | 1170012 | 55166 | 1.93E+09 | 8.82E+09 | 4.85E+09 |
| Boston | MA-NH | 5327337 | 320435 | 9.77E+09 | 9.02E+09 | 1.55E+10 |
| Buffalo | NY | 1230213 | 46483 | 3.59E+09 | 4.17E+09 | 4.23E+09 |
| Charleston | SC | 594899 | 27551 | 5.09E+08 | 7.39E+08 | 1.40E+09 |
| Charlotte | NC-SC | 1834307 | 116610 | 5.23E+09 | 1.23E+10 | 7.63E+09 |
| Chicago | IL-IN-WI | 9501296 | 553309 | 1.95E+10 | 3.09E+10 | 3.69E+10 |
| Cincinnati | OH-KY-IN | 1631243 | 105377 | 2.46E+09 | 8.46E+09 | 7.29E+09 |
| Cleveland | OH | 2931774 | 112443 | 2.70E+09 | 4.26E+09 | 2.42E+09 |
| Columbus | OH | 1936351 | 97846 | 3.65E+09 | 1.41E+10 | 7.87E+09 |
| Corpus Christi | TX | 444727 | 17663 | 1.80E+09 | 1.46E+09 | 5.87E+09 |
| Dallas | TX | 6171301 | 360344 | 2.87E+10 | 3.29E+10 | 2.97E+10 |
| Dayton | OH | 1078634 | 36875 | 2.48E+09 | 5.78E+09 | 1.60E+10 |
| Denver | CO | 2869377 | 148700 | 1.18E+10 | 2.84E+10 | 1.44E+10 |
| Detroit | MI | 5428000 | 221559 | 6.11E+09 | 5.82E+09 | 5.45E+09 |
| El Paso | TX | 721598 | 25757 | 1.13E+09 | 1.77E+09 | 6.27E+08 |
| Grand Rapids | MI | 1315319 | 44148 | 5.13E+09 | 6.33E+09 | 5.14E+09 |
| Greensboro | NC | 1490886 | 34801 | 2.25E+09 | 8.00E+09 | 4.03E+09 |
| Greenville | SC | 1185534 | 31607 | 9.34E+08 | 2.71E+09 | 2.87E+09 |
| Hartford | CT | 1304067 | 85402 | 1.48E+09 | 2.05E+09 | 1.41E+09 |
| Honolulu | HI | 905266 | 153098 | 2.94E+09 | 7.88E+08 | 2.64E+08 |
| Houston | TX | 5380661 | 376251 | 2.12E+10 | 2.92E+10 | 2.07E+10 |
| Indianapolis | IN | 1958453 | 109728 | 5.50E+09 | 2.08E+10 | 1.74E+10 |
| Jacksonville | FL | 1277763 | 62765 | 1.94E+09 | 8.77E+09 | 4.03E+09 |
| Kansas City | MO-KS | 2015282 | 105723 | 5.05E+09 | 2.13E+10 | 2.95E+10 |
| Lake Charles | LA | 226249 | 12559 | 6.85E+08 | 8.33E+08 | 7.50E+08 |
| Laredo | TX | 224695 | 5889 | 3.73E+08 | 2.64E+09 | 2.17E+08 |
| Las Vegas | NV | 1751028 | 98836 | 3.49E+09 | 3.57E+09 | 2.76E+08 |
| Los Angeles | CA | 17629607 | 781556 | 5.72E+10 | 4.05E+10 | 4.24E+10 |
| Louisville | KY-IN | 1080797 | 58722 | 1.27E+09 | 7.51E+09 | 4.17E+09 |
| Memphis | TN-MS-AR | 999491 | 66097 | 2.03E+09 | 6.34E+09 | 7.18E+09 |
| Miami | FL | 5428962 | 277895 | 4.64E+10 | 8.36E+09 | 2.73E+10 |
| Milwaukee | WI | 1708563 | 86967 | 3.96E+09 | 5.36E+09 | 4.63E+09 |
| Minneapolis-St. Paul | MN-WI | 3350862 | 199702 | 7.47E+09 | 4.35E+10 | 3.99E+10 |
| Mobile | AL | 564013 | 16002 | 1.19E+09 | 1.76E+09 | 7.47E+08 |
| Nashville | TN | 1498836 | 81223 | 3.82E+09 | 1.25E+10 | 4.91E+09 |
| New Orleans | LA | 1363990 | 67812 | 2.15E+09 | 6.86E+10 | 2.40E+10 |
| New York | NY-NJ-CT-PA | 21847286 | 1311356 | 3.31E+10 | 2.79E+10 | 2.41E+10 |
| Norfolk | VA-NC | 1624234 | 153098 | 2.38E+09 | 5.62E+09 | 7.42E+09 |
| Oklahoma City | OK | 1223100 | 55510 | 4.41E+09 | 2.39E+10 | 1.32E+10 |
| Orlando | FL | 2600672 | 110089 | 3.78E+09 | 7.50E+09 | 2.93E+09 |
| Philadelphia | PA-NJ-DE-MD | 5751995 | 341474 | 5.85E+09 | 1.25E+10 | 1.22E+10 |
| Phoenix | AZ | 3865077 | 202798 | 3.25E+10 | 1.57E+10 | 1.76E+10 |
| Pittsburgh | PA | 2478883 | 114151 | 2.39E+09 | 4.15E+09 | 2.63E+09 |
| Portland | OR-WA | 1681431 | 120599 | 6.21E+09 | 2.11E+10 | 2.94E+10 |
| Raleigh-Durham | NC | 1509560 | 55834 | 3.01E+09 | 6.43E+09 | 7.24E+09 |
| Richmond | VA | 1175654 | 61790 | 2.02E+09 | 5.16E+09 | 6.10E+09 |
| Rochester | NY | 1133140 | 48923 | 3.10E+09 | 3.37E+09 | 4.22E+09 |
| Sacramento | CA-NV | 2296706 | 103119 | 5.93E+09 | 1.32E+10 | 1.69E+10 |
| Salt Lake City | UT | 1632482 | 64435 | 6.04E+09 | 8.75E+09 | 7.74E+09 |
| San Antonio | TX | 1889797 | 77723 | 4.94E+09 | 9.95E+09 | 8.17E+09 |
| San Diego | CA | 2933462 | 181424 | 5.01E+09 | 3.75E+09 | 3.82E+09 |
| San Francisco | CA | 7168176 | 332416 | 1.74E+10 | 2.41E+10 | 1.42E+10 |
| Savannah | GA | 382510 | 13888 | 2.81E+08 | 7.44E+08 | 2.25E+09 |
| Seattle | WA | 3919624 | 244186 | 1.23E+10 | 2.45E+10 | 2.96E+10 |
| St. Louis | MO-IL | 2840179 | 132912 | 8.07E+09 | 2.44E+10 | 2.61E+10 |
| Tampa | FL | 2636972 | 119518 | 7.53E+09 | 9.45E+09 | 1.42E+10 |
| Tucson | AZ | 924786 | 35414 | 1.40E+09 | 2.49E+09 | 1.24E+09 |
| Tulsa | OK | 934820 | 44328 | 2.58E+09 | 6.23E+09 | 4.21E+09 |
| Washington DC | DC-VA-MD-WV | 5430278 | 405721 | 3.65E+09 | 9.08E+09 | 5.41E+09 |

**References:**

1. Southworth F, Peterson BE, Hwang HL, Chin SM, Davidson D. The Freight Analysis Framework Version 3 (FAF3): A Description of the FAF3 Regional Database and How It Is Constructed. Oak Ridge, Tennessee. 2011.
2. Mekonnen MM, Hoekstra AY. The green, blue and grey water footprint of crops and derived crop products. Hydrology and Earth System Sciences. 2011 May 1;15(5):1577.
3. Mubako ST. Frameworks for estimating virtual water flows among US states. Southern Illinois University at Carbondale; 2011.
4. USDA. Quick Stats. 2007. <https://quickstats.nass.usda.gov/>, accessed July 2017.
5. CDM-Smith. IWR-MAIN Water Use Coefficients. 1996.
6. Ahams IC, Paterson W, Garcia S, Rushforth R, Ruddell BL, Mejia A. Water Footprint of 65 Mid‐to Large‐Sized US Cities and Their Metropolitan Areas. JAWRA Journal of the American Water Resources Association. 2017 Oct 1;53(5):1147-63.
7. Kenny JF, Barber NL, Hutson SS, Linsey KS, Lovelace JK, Maupin MA. Estimated use of water in the United States in 2005. US Geological Survey; 2009.
8. Solley WB, Pierce RR, Perlman HA. Estimated use of water in the United States in 1995. US Geological Survey; 1998.
9. U.S. Census Bureau. County and City Data Book: 2007 (14^th^ Edition), Washington, D.C.
10. BEA, 2007. Regional Economic Accounts. <http://www.bea.gov/regional/index.htm>, accessed July 2017.
